# Promotion of hydrogen evolution from seawater via poly(aniline-co-4-nitroaniline) combined with 3D nickel nanoparticles

**DOI:** 10.1038/s41598-023-48355-3

**Published:** 2023-12-06

**Authors:** Saleh Moradi-Alavian, Amir Kazempour, Meysam Mirzaei-Saatlo, Habib Ashassi-Sorkhabi, Abbas Mehrdad, Elnaz Asghari, Jacob J. Lamb, Bruno G. Pollet

**Affiliations:** 1 Electrochemistry Research Laboratory, Department of Physical Chemistry, Faculty of Chemistry, University of Tabriz, Tabriz, Iran; 2 Department of Physical Chemistry, Faculty of Chemistry, University of Tabriz, Tabriz, Iran; 3 Hydrogen Energy and Sonochemistry Research Group, Department of Energy and Process Engineering, Faculty of Engineering, Norwegian University of Science and Technology (NTNU), NO-7491 Trondheim, Norway; 4 Department of Energy and Process Engineering & ENERSENSE, Norwegian University of Science and Technology (NTNU), NO-7491 Trondheim, Norway; 5 Green Hydrogen Lab, Institute for Hydrogen Research (IHR), Université du Québec à Trois-Rivières (UQTR), 3351 Boulevard des Forges, Trois-Rivières, QC G9A 5H7 Canada

**Keywords:** Chemistry, Electrochemistry, Energy, Physical chemistry

## Abstract

This work reports the synthesis of poly (aniline-co-4-nitroaniline) deposited on a three-dimensional nanostructured nickel (3D-Ni) film, where both layers were fabricated via potentiostatic electrodeposition. The obtained electrocatalyst exhibited excellent electrochemical activity for the Hydrogen Evolution Reaction (HER) with small overpotentials of − 195 and − 325 mV at − 10 and − 100 mAcm^−2^, respectively, and a low Tafel slope of 53.3 mV dec^−1^ in seawater. Additionally, the electrocatalyst exhibited good stability after 72 h operation under a constant potential of − 1.9 V vs*.* RHE. The efficient HER performance of the *as*-prepared catalyst was found to originate from the synergy between the conducting polymer and three-dimensional nickel nanoparticles with a large electrochemical active surface area. Moreover, the results obtained from electrochemical impedance spectroscopy (EIS) measurements revealed that the presence of 3D-Ni layer improved the kinetics of HER by reducing the charge transfer resistance for the electrocatalyst.

## Introduction

Because of its numerous advantages, including zero carbon emissions, exceptional efficiency, and mobility, electrochemically derived hydrogen gas is now considered one of the most promising sustainable energy storage forms for a variety of applications^1–5^. Water electrolysis is regarded as an efficient method for producing hydrogen in large quantities and with high purity due to its simplicity. In terms of the electrolysis of water, it is necessary to lower electrode reaction overpotentials and choose affordable electrode materials with high electrocatalytic activity. Within this framework, several catalysts have been investigated as potential electrode materials for the creation of renewable hydrogen. Due to their high electrocatalytic impact and low overpotentials for the Hydrogen Evolution Reaction (HER), most noble metals (in particular Pt), were the top choice materials^6–11^. Despite this, their exorbitant price and global scarcity have limited their vast usage. As a result, it appears crucial to synthesize materials suitable for the catalysis of HER in place of Pt. Conducting polymers might be an attractive choice, given their capacity to enhance the electrochemical characteristics of electrodes in a variety of applications, including electrocatalysis, carbon dioxide electrochemical reduction, and corrosion protection^12–15^. Polyaniline (Pani) and its derivatives are particularly well suited for usage as an appropriate electrocatalyst due to their high conductivity, environmental stability, chemical and physical characteristics, ease of synthesis, lightweight, and creation from affordable monomers^16–20^. A Pani derivative called poly (aniline-co-4-nitroaniline) has also been used in a few polymer solar cell applications^21,22^. The optical, electronic, and catalytic properties of this polymer have also been investigated^23–25^. Moreover, earlier studies have shown the ability of a three-dimensional nickel nanoparticle layer to catalyze the HER. For example, by heat treating the electrodeposited Zn layer on a nickel substrate and then electrochemically dealloying, a three-dimensional nanoporous nickel film was created^26^. A three-dimensional nickel-carbon composite was also generated by the electrodeposition approach^27,28^. Two distinct three-dimensional nickel composites containing 50 nm carbon particles and activated carbon were effectively synthesized, and their HER-activating properties were assessed^29^. All these studies showed that three-dimensional nickel-included catalysts had good performance in the HER. This prompted us to create a novel catalyst with much increased catalytic activity by combining such a structure with conducting polymers. As far as we are aware, there is no information about the catalytic properties of three-dimensional nickel films linked to poly (aniline-co-4-nitroaniline). Considering this, we anticipate that the results of this work will be useful in the development of affordable and effective HER catalysts.

## Experimental

### Preparation of electrodes

To create the deposition solution, 2.87 g of NiSO_4_.6H_2_O (99%, Fluka), 10.70 g of NH_4_Cl (99.8%, Merck), and 11.70 g of NaCl (99.5%, Merck) were dissolved in 100 mL of distilled water to create the solution for the 3D nanostructured Ni (3D-Ni) film. A nickel plate (1 × 1 cm^2^), a graphite sheet, and an Ag/AgCl (Sat. KCl) electrode were used as the working, counter, and reference electrodes, respectively, to electrodeposit the 3D-Ni film at a constant potential of − 5 V for 90 s. A MIRA3 TESCAN field emission scanning electron microscope (FESEM) and a Philips X-ray diffractometer (XRD) were used to analyze the produced Ni layer.

To synthesize poly (aniline-co-4-nitroaniline), simply defined as 4-NPani, a 0.5 M H_2_SO_4_ solution containing 0.1 M aniline (99%, Merck) and various concentrations of 4-nitroamiline (0.00, 0.25, 0.50, 1.00, and 2.00 mM) was prepared. The deposition of 4-NPani onto the as-prepared 3D-Ni electrode was carried out using the potentiostatic method at three different potentials of 0.72, 0.76, and 0.80 V. Different deposition times, in the range of 100 to 400 s, were also examined to observe how they affected the electrode characteristics. The 4-NPani film was characterized using field emission scanning electron microscopy (FE-SEM) and Fourier transform infrared spectroscopy (FT-IR) spectroscopy (Tensor 27 Bruker). After deposition of 4-NPani onto the 3D-Ni layer, the whole electrode was subjected to HER experiments in a simulated seawater environment.

### Electrochemical measurements

Cathodic polarization from 0.0 to − 2.0 V at a scan rate of 2.0 mV s^–1^ was used to generate LSV diagrams that considered the *iR* drop correction. The EIS measurement was carried out under a sinusoidal signal with an amplitude of ± 10 mV and a frequency range of 100 kHz to 0.01 Hz. The ZView (II) software was used to generate an equivalent circuit model to determine the EIS parameter values. Chronoamperometry was also performed to evaluate the stability of the electrode over a 3-day period with a constant potential of − 1.9 V vs*.* RHE. All measurements were carried out in a conventional three-electrode cell by an AUTOLAB PGSTAT30 potentiostat–galvanostat. Ag/AgCl (Sat. KCl) served as the reference electrode, and a graphite sheet with a surface area of about 3 cm^2^ served as the counter electrode. The electrolyte used for the measurements was artificial seawater with a pH of 7.0, which had a composition of NaCl (38.38 g L^–1^), CaCl_2_ (2.43 g L^–1^), MgCl_2_ (19.06 g L^–1^), Na_2_SO_4_ (5.26 g L^–1^), and KHCO_3_ (0.24 g L^–1^).

## Results and discussion

### Characterizations

**Figure 1:**
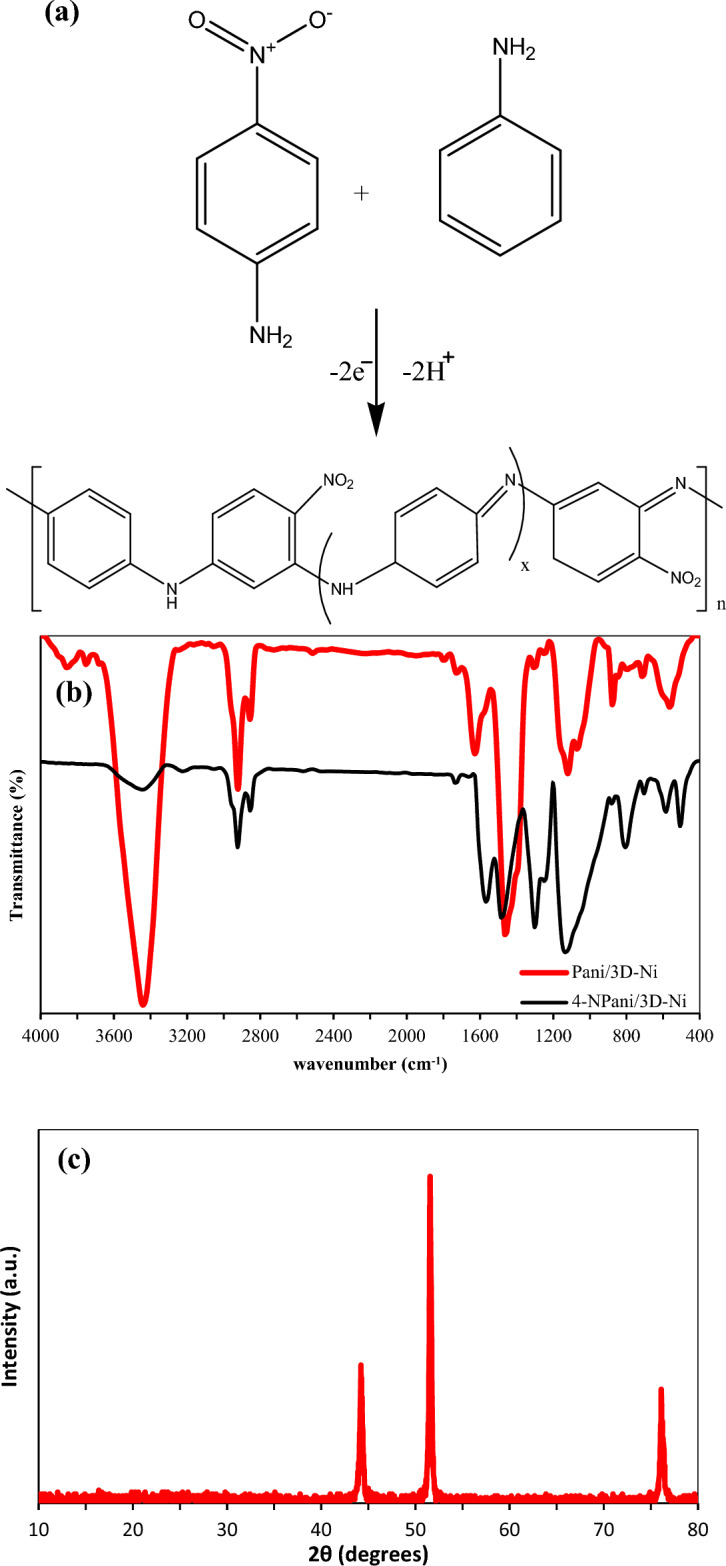
(**a**) Chemical reaction of electrodeposited 4-NPani/3D-Ni, (**b**) FT-IR spectra of synthesized Pani/3D-Ni and 4-NPani/3D-Ni, and (**c**) XRD pattern of the electrodeposited 3D-Ni.

It is simple to create 4-NPani/3D-Ni via electrochemical polymerization when aniline and 4-nitroaniline are combined. Figure 1a illustrates the suggested electrochemical synthesized mechanism of 4-NPani.

Figure 1b exhibits the FT-IR spectra of electrodeposited 4-NPani/3D-Ni and Pani/3D-Ni electrodes. Accordingly, the stretching vibration of the –NH group caused a wide peak to form at 3440 cm^–1^ (Pani has –NH groups in its structure more than 4-NPani so it showed high transmittance). The asymmetric stretching and symmetric stretching vibrations of the C–H groups were attributed to the bands seen at 2924 and 2855 cm^–1^, respectively. For copolymers and polyanilines, the bands at 1623 and 1464 cm^–1^ are indicative of C=C quinonoid and benzenoid stretching vibrations, respectively. The stretching vibration of C–N was observed at 1475 and 1460 cm^–1^. A band at 1297 cm^–1^ was caused by C–N in-plane bending vibration. The C–H stretching vibration bands appeared at 2856 and 2884 cm^–1^, and the N–O stretching vibration of the copolymer had bands at 1579 and 1327 cm^–1^. The 1, 2, 4 tri-substitution across the benzene ring was responsible for the weak bands detected at 794 and 1153 cm^–1^^21,22,25^. This demonstrated that the 4-nitroaniline monomer was successfully incorporated into the polymer backbone.

XRD analysis was used to analyze the synthesized 3D-Ni layer, as illustrated in Fig. 1c. Diffraction peaks at 44.2°, 51.5°, and 76.1° were an indication of the production of nickel crystalline structures^30,31^.

**Figure 2:**
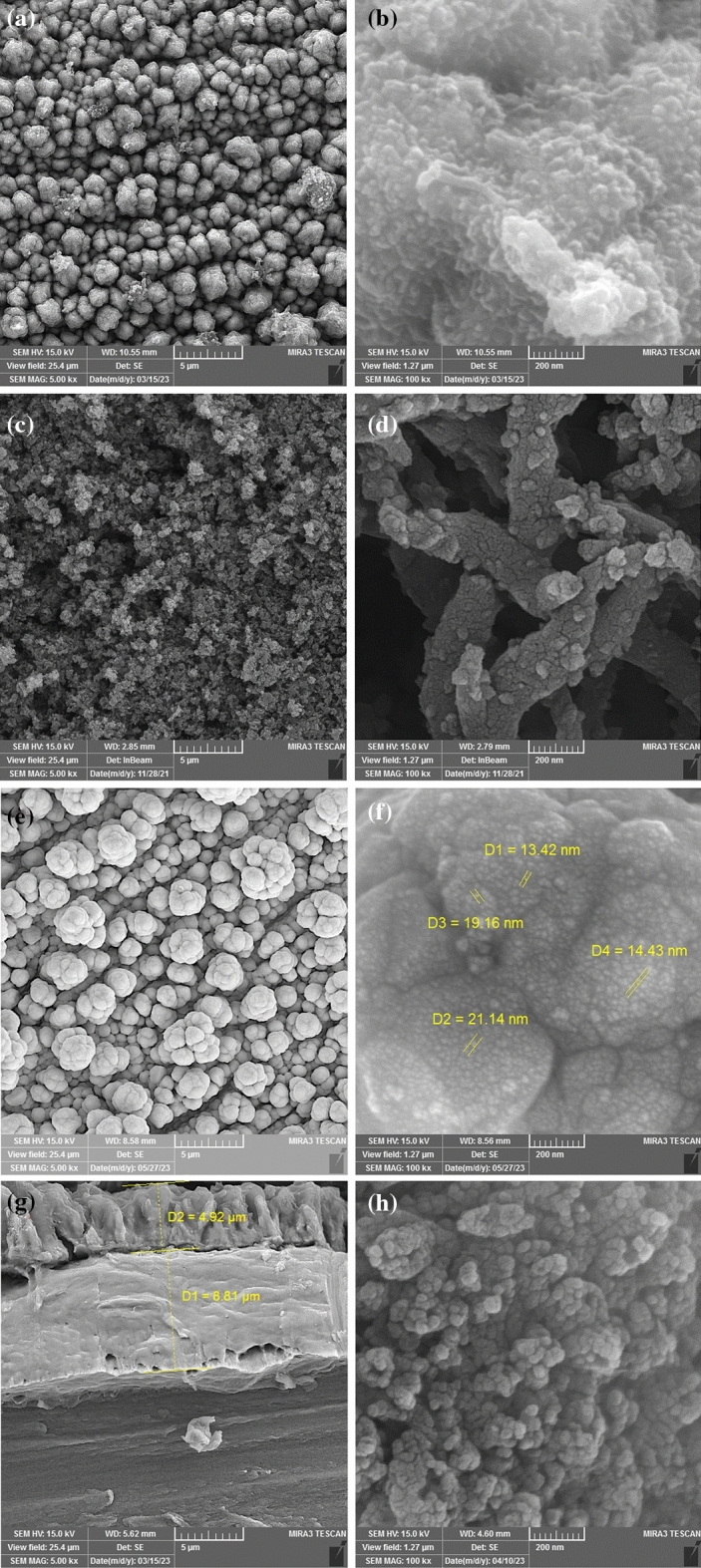
FE-SEM images of the *as*-prepared electrodes (**a**,**b**) 4-N Pani/3D-Ni, (**c**,**d**) Pani/3D-Ni, (**e**,**f**) 3D-Ni, (**g**) cross section of 4-N Pani/3D-Ni, and (**h**) 4-N Pani/3D-Ni after 72 h electrolysis in seawater at − 1.9 V vs. RHE.

The SEM images of 4-N Pani/3D-Ni, Pani/3D-Ni, and 3D-Ni are depicted in Fig. 2. According to these images, Fig. 2a and b show a uniform spherical structure for the synthesized 4-N Pani/3D-Ni with an average size of 1–2 µm. On the other hand, Pani/3D-Ni illustrates a worm-like structure that was completely different from the 4-N Pani/3D-N structure (Fig. 2c and d). The SEM images related to 3D-Ni showed a structural difference between the uniformly synthesized 3D-Ni and its top layers (Fig. 2e and f). This figure demonstrated a cauliflower-like structure for 3D-Ni with an average size of 2–4 µm, while the grain size of Ni nanoparticles were in the range of 15 to 25 nm. Furthermore, the 4-N Pani/3D-Ni coating displayed a morphology that was different from that of Pani/3D-Ni coating (Fig. 2c and d). A comparison between the morphologies of 4-N Pani/3D-Ni and Pani/3D-Ni (Fig. 2c and d) coatings revealed that the surface of 4-N Pani/3D-Ni had a spherical structure, whereas Pani/3D-Ni had a worm-like structure. A cross-section SEM image, shown in Fig. 2g, was used to determine the thickness of the 4-N Pani/3D-Ni coating. This image depicted two layers: the top layer was related to the 4-NPani coating with a thickness of about 4.92 µm, and the middle one corresponded to the 3D-Ni coating with a thickness of around 8.81 µm. Figure 2h shows the surface of 4-NPani/3D-Ni electrode after 72 h of electrolysis at − 1.9 V vs. RHE. According to this figure, some particles of the polymer that were loosely attached to the electrode surface separated from the surface. This caused a slight change in the coating morphology, increasing the surface area of the electrode.

### Electrochemical results

**Figure 3:**
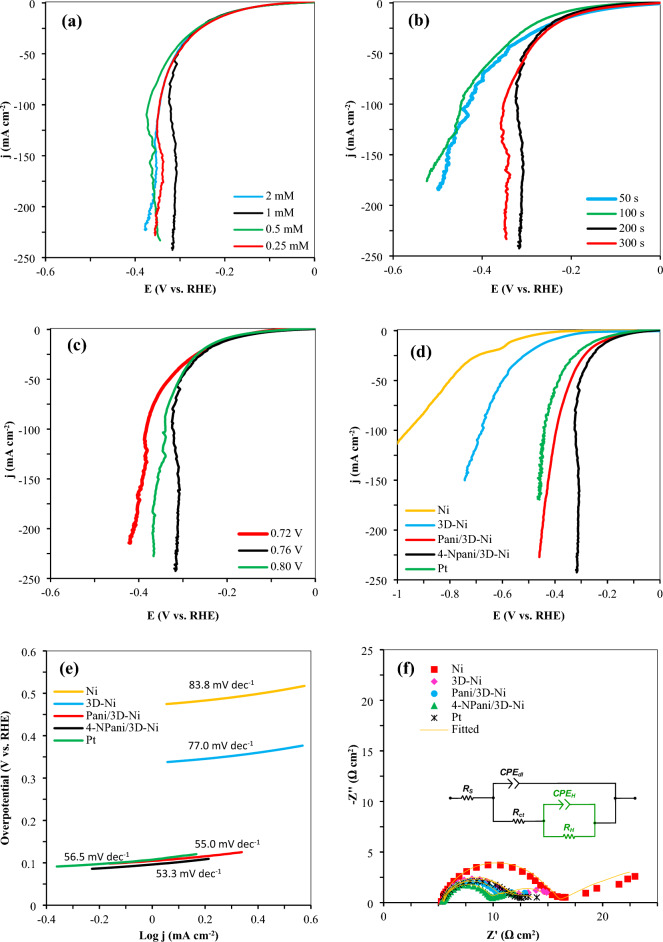
The electrochemical performance of various fabricated electrodes in seawater at room temperature (**a**) LSV plots of the synthesized 4-N Pani/3D-Ni with different concentrations of 4-nitroaniline at a potential of + 0.76 V vs. RHE, (**b**) LSV plots of the synthesized 4-NPani/3D-Ni at various electrodeposition times under a potential of + 0.76 V vs*.* RHE, (**c**) LSV plots of the synthesized 4-NPani/3D-Ni at different potentials (**d**) LSV plots for comparing the performance of various prepared electrodes, (**e**) Tafel slope diagrams for the prepared electrodes, and (**f**) EIS analyses of the electrodes in seawater at a potential of − 0.6 V vs*.* RHE, where the inset figure shows the equivalent circuit model applied for fitting the EIS plots.

The investigation of various electrodeposition circumstances led to the development of a functioning electrode with the maximum activity. Various parameters, including potential and time of depositions, as well as 4-nitroaniline concentration, were optimized to fabricate this electrode. Moreover, different concentrations (0.25, 0.5, 1.00, and 2.00 mM) from this material were added to the aniline solution to reach an optimum concentration of 4-nitroaniline, and 1 mM of 4-nitroaniline had the best performance as a catalyst of HER as it is obvious from the LSV plots in Fig. 3a. The optimum electrodeposition time and potential were also obtained as 200 s and 0.76 V vs. RHE, as demonstrated in Fig. 3b and c, respectively. The best electrode (4-N Pani/3D-Ni) was compared to those of the blank Ni, 3D-Ni, Pani/3D-Ni, and Pt as a favorable electrode for the HER as shown by the LSV diagram (Fig. 3d). The unique porous structure of the 3D-Ni coating resulted in higher electrocatalytic activity (i.e. lower overpotential and higher current density) than the blank Ni (Fig. 3d). The foam-like nature of the 3D-Ni increased the active surface area, which contributed to the HER. As seen in Fig. 3d, the presence of 4-NPani copolymer significantly improved the HER activity of the 3D-Ni electrode. This improvement allowed the performance of 4-NPani/3D-Ni electrode to be even better than that of pure Pt. As shown in Fig. 3d, 4-NPani/3D-Ni required very low overpotentials of − 195 and − 325 mV to reach current densities of − 10 and − 100 mA cm^-2^, respectively. These values were substantially less than those of the Ni (− 558 and − 959 mV), 3D-Ni (− 417 and − 676 mV), Pani/3D-Ni (− 212 and − 395 mV), and Pt (− 244 and − 441 mV).

**Figure 4:**
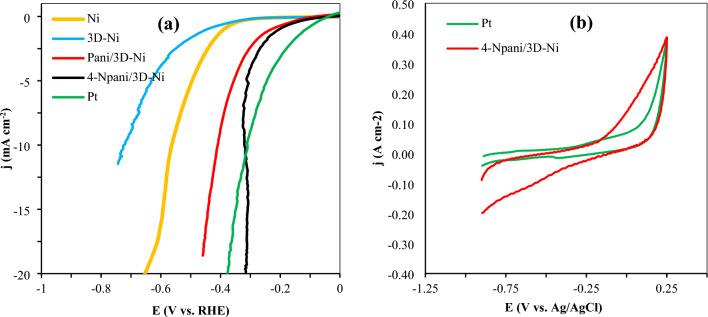
(**a**) LSV diagrams of the studied electrodes after normalizing by ECSA reported in Table 2, and (**b**) cyclic voltammetry diagrams of Pt and 4-NPani/3D-Ni in sea water at scan rate of 50 mV s^–1^ to obtain TOF values.

Tafel slope values were used to evaluate the intrinsic catalytic kinetics of the electrodes (Fig. 3e). The Tafel slope (b) was derived from *η* = a + b log (*j*), where *η* is the overpotential and *j* is the current density. The Tafel slope offers details on the reaction process. In this work, procedures including applying a low scan rate for LSV measurements and *iR* drop correction were taken into consideration to provide Tafel and LSV analyses that were more accurate. As seen in Fig. 3e, 4-N Pani/3D-Ni had a low Tafel slope value of 53.3 mV dec^–1^, being slightly less than those of Pani/3D-Ni (55.0 mV dec^–1^) and Pt (56.5 mV dec^–1^), and also much lower than those of Ni (83.3 mV dec^–1^) and 3D-Ni (77.0 mV dec^–1^). This observation implied a faster charge transfer and in turn improved catalytic kinetics for 4-NPani/3D-Ni electrode^32–34^. Table 1 illustrates some results from the literature, and it can be seen that the 4-NPani/3D-Ni electrode showed proper performance. Figure 4a depicts the LSV diagrams of all the studied electrodes after normalizing via electrochemical active surface areas (*ECSA*) obtained from EIS data (see Table 2). This figure showed that the 4-NPani/3D-Ni electrode had a catalytic activity comparable to Pt even after normalizing the LSV curves. Additionally, turnover frequency (TOF) of the two electrodes Pt and 4-NPani/3D-Ni was calculated according to the literature^35–38^. For this purpose, CV diagrams were provided in a potential range of − 0.90 to 0.25 in seawater, as shown in Fig. 4b. TOF is a measure of the number of H_2_ molecules generated from the electrode surface per unit second. The TOF values of Pt and 4-NPani/3D-Ni were estimated 5.30 and 14.50 s^–1^, respectively.

**Table 1:** Comparison of some finding from the literature with the results of this study.

Catalyst	Electrolyte	*η* (mV)10 mA cm^–2^	Tafel slope (mV dec^–1^)	Stability	Ref
PSS-PPy/Ni-Co-P	PBS (1M)	106	80.81	90 h	^39^
PSS-PPy/Ni-Co-P	KOH (1M)	67	27.38	24 h	^39^
WO3@NPRGO	H_2_SO_4_ (0.5M)	225	87	1000 cycles	^40^
Co(II)-PPy NPs/NF	NaOH(1M)	–	110	30 h	^41^
PTh:PPP	KOH (1M)	77	152	–	^42^
Ni-PAni 175	H_2_SO_4_ (0.5M)	82	131	–	^43^
4-NPani/3D-Ni	Sea water	195	53.3	72 h	This work

**Table 2:** EIS diagrams fitting parameters of prepared electrodes in seawater at a potential of − 0.6 V vs RHE at room temperature.

Samples	*CPE*_dl_		*R*_ct_ (Ω cm^2^)	*CPE*_H_		*R*_H_ (Ω cm^2^)	*C*_dl_ (mF)	*ECSA* (cm^2^)	Fitting error
	*Y*_0_ × 10^–3^ (Ω^–1^ cm^–2^S^n^)	*n*		*Y*_0_ (Ω^–1^ cm^–2^S^n^)	*n*				
Ni	0.134	0.85	9.770	0.102	0.53	14.76	0.04	1.06	0.0047
3D-Ni	0.965	0.89	5.990	0.109	0.81	3.80	0.51	13.09	0.0019
Pt	0.248	0.80	5.880	0.060	0.52	2.96	0.05	1.24	0.0016
Pani/3D-Ni	1.330	0.83	5.012	0.180	0.76	3.14	0.48	12.22	0.0002
4-NPani/3D-Ni	1.216	0.84	4.450	0.202	0.80	2.58	0.45	11.54	0.0013

EIS might be used to investigate the HER kinetics and the reactions at the electrode/electrolyte interface. Figure 3f depicts the Nyquist diagrams of the electrodes in seawater at a potential of − 0.6 V vs RHE. According to this figure, the Nyquist diagrams demonstrated the presence of two-time constants. The first-time constant was associated with charge transfer kinetics, whereas the second one was related to the hydrogen adsorption process. As a result, it was predicted that impedance would produce data that was compatible with steady-state observations. The Electrical Equivalent Circuit (EEC) model illustrated in the inset of Fig. 3f was used to analyze the obtained EIS data. This model represents the behavior of the HER system^44^, where the solution resistance is *R*_s_, the adsorption constant phase element is *CPE*_H_, and the adsorption resistance is *R*_H_. Additionally, *CPE*_dl_ denotes the double-layer constant phase element, and *R*_ct_ denotes the electrode's interface charge-transfer rate. *R*_ct_ is mostly determined by the intrinsic features of the catalyst material, where a low *R*_ct_ value suggests a quick reaction rate. It is known that a lower value of charge transfer resistance is associated with the higher electrocatalytic activity of electrodes for hydrogen generation^10^. The charge-transfer mechanism is broadly represented by a master equation from Volmer-Heyrovsky, which represents the widely accepted reaction mechanism for the HER in alkaline conditions^45^. Moreover, the adsorption resistance (*R*_H_) is represented by the semi-circle in the low-frequency zone, and since *R*_H_ represents the HER onset potential, a low *R*_H_ value denotes a quickly occurring onset of HER^46^. To explain the non-ideal performance of solid electrodes, Constant Phase Elements (*CPE*) are utilized instead of ideal capacitors. *CPE* consists of two elements, *Y*_0_ and *n*, where *Y*_0_ is the *CPE* constant and *n* is a *CPE* exponent that may be used to quantify surface heterogeneity or roughness^47^. The various *CPE* parameter amounts found in our studies might be attributed to electrode grain surface dispersion. Indeed, stochastic bulk distributions of grain forms, sizes, and orientations are present in electrodes, causing stochastic grain distributions to arise at the electrode^48^. It is typically challenging to demonstrate how much hydrogen has been adsorbed onto the electrode surface when there are bubbles present. The hydrogen bubble growth caused by applied overpotential on the surface of electrodes is commonly regarded as a source of noise that results in some fluctuations in impedance experimental results especially at low frequencies (the hydrogen adsorption relaxation loop)^49^. The relaxation will be influenced by the electrolyte in contact with the electrodes as well as the physical characteristics of the electrodes^49,50^.

Table 2 listed the quantitative data that were obtained by fitting EIS diagrams. This table revealed that the *R*_ct_ values of the investigated electrodes were in the following order: Ni > 3D-Ni > Pt > Pani/3D-Ni > 4-NPani/3D-Ni. According to this finding, 4-NPani/3D-Ni transferred electrons across its surfaces quicker than the other electrodes. This decrease in resistance resulted from the fact that embedding an electronic conducting layer would reduce the catalytic system's charge transfer resistance. Additionally, coating the Ni blank with a 3D-Ni layer that has a large active surface area and high conductivity lowered the *R*_ct_. The order observed in the *R*_H_ values of the samples revealed that 4-NPani/3D-Ni had the lowest *R*_H_, meaning a facile hydrogen adsorption to the surface of 4-NPani/3D-Ni electrode. A low charge transfer resistance obtained for the 3D-Ni layer suggested that this layer was helpful for gaining a faster HER kinetics^51–53^. The values of *C*_dl_ can be used to estimate the ECSA values of the electrodes by using the following equation *ECSA* = *C*_dl_/*C*_s_, where *C*_s_ is general specific capacitance. 0.039 mF cm^-2^ of general specific capacitance was used for Pt and Ni substrates^54–58^. Equation (1) was also used to compute the values of double-layer capacitance^59^, and the results are given in Table 2.

1 $$C\mathrm{dl}={({Y}_{0}\times {R}_{ct})}^\frac{1}{n}/{R}_{ct}.$$

Based on the data in Table 2, the *ECSA* values of the metals (blank Ni and Pt) were significantly lower than those of the porous and nanostructured electrodes, suggesting that the rigid surface of blank Ni and Pt was responsible for their lower *ECSA* values.

**Figure 5:**
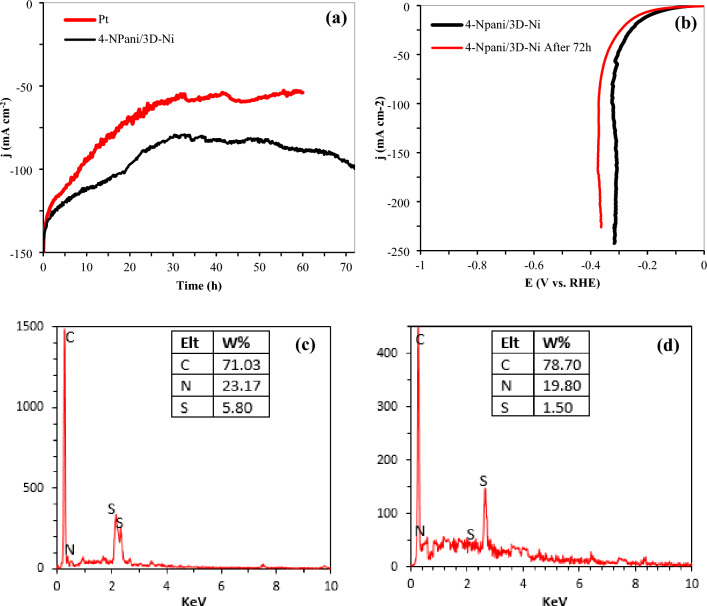
(**a**) Chronoamperometry for 4-N Pani/3D-Ni and Pt electrodes at a constant applied potential of -1.9 V vs. RHE, (**b**) LSV diagram of 4-N Pani/3D-Ni before and after long-term stability test, (**c**) EDX analysis of 4-N Pani/3D-Ni before stability test, and (**d**) EDX analysis of 4-N Pani/3D-Ni after stability test.

In real-time applications, the functional stability of a coating for HER is more significant than the catalytic activity^60^. The long-term stability of 4-N Pani/3D-Ni was tested at a constant voltage of − 1.9 V vs. RHE in seawater for 72 h. Figure 5a depicts the stability of 4-N Pani/3D-Ni and Pt electrodes for 72 h and 60 h, respectively. According to this figure, 4-N Pani/3D-Ni exhibited better performance than Pt, meaning that a lower potential input was required for 4-N Pani/3D-Ni to generate hydrogen from seawater. It was found that the current densities of 4-N Pani/3D-Ni and Pt decreased until 34 h of electrolysis, then Pt was almost stable while the current density of 4-N Pani/3D-Ni increased. These observations could be due to the poisoning of the Pt active sites by the adsorbed chloride ions (Cl^-^), blocking the absorption of hydrogen atoms^61^. Additionally, Fig. 5b compares the LSV diagrams of 4-N Pani/3D-Ni before and after electrolysis. Accordingly, the HER activity of the electrode did not show a significant redaction after 72 h of electrolysis in sea water. This conclusion could be supported by the EDX analysis represented in Fig. 5c and d, indicating that the electrode composition did not undergo a remarkable change after electrolysis. Furthermore, the improved performance of 4-N Pani/3D-Ni after 34 h of electrolysis could be due to the increased surface area of the electrode, as demonstrated by the SEM images shown in Fig. 2h.

## Conclusions

Linear sweep voltammogram experiments for Pani/3D-Ni and 4-N Pani/3D-Ni showed that the addition of NO_2_ groups derived from 4-nitroaniline to the Pani structure improved the catalytic activity of the resultant electrodes. A remarkable synergism between 3D-Ni and 4-N Pani led to higher HER performance. It was also found that the Tafel slopes decreased from 77 mV dec^–1^ for the 3D-Ni electrode to 53 mV dec^–1^ of 4-N Pani/3D-Ni electrode, a value comparable to that found for Pt (56 mV dec^–1^). The improved catalytic activity for 3D-Ni was mainly due to the increased electrochemical active surface area. Long-term durability studies of the *as*-prepared electrodes immersed in seawater for 3 days were performed by chronoamperometry and showed that the electrode was stable with an improvement in its activity. Overall, the promising results found for 4-N Pani/3D-Ni indicated that it can be used for efficient hydrogen evolution in seawater when compared to Pt.

## Data Availability

The datasets used and/or analysed during the current study available from the corresponding author on reasonable request.

## References

[1] Singh R, Singh M, Gautam S (2021). Hydrogen economy, energy, and liquid organic carriers for its mobility. Mater. Today Proc..

[2] Mahlia TMI, Saktisahdan TJ, Jannifar A, Hasan MH, Matseelar HSC (2014). A review of available methods and development on energy storage; technology update. Renew. Sustain. Energy Rev..

[3] Kaur M, Pal K (2019). Review on hydrogen storage materials and methods from an electrochemical viewpoint. J. Energy Stor..

[4] Sazali N (2020). Emerging technologies by hydrogen: A review. Int. J. Hydrogen Energy.

[5] Li J (2023). Structure phase engineering strategy through acetic acid coupling to boost hydrogen evolution reaction performance of 2H phase MoS_2_ at wide pH range. Fuel.

[6] Hussain N, Alawadhi H, Olabi AG, Elsaid K, Abdelkareem MA (2023). Construction of Cu_2_O-g-C_3_N_4_/MoS_2_ composite material through the decoration of Cu_2_O nanoparticles onto the surface of two-dimensional g-C_3_N_4_/MoS_2_ heterostructure for their application in electrochemical hydrogen evolution. Fuel.

[7] Manzotti A (2022). Membraneless electrolyzers for the production of low-cost, high-purity green hydrogen: A techno-economic analysis. Energy Convers. Manag..

[8] Paygozar S, Aghdam ASR, Hassanizadeh E, Andaveh R, Darband GB (2023). Recent progress in non-noble metal-based electrocatalysts for urea-assisted electrochemical hydrogen production. Int. J. Hydrogen Energy.

[9] Al-Hamamre Z, Karimzadeh Z, Ji S, Choi H, Maleki H (2022). Aerogels-inspired based photo and electrocatalyst for water splitting to produce hydrogen. Appl. Mater. Today.

[10] Tan Y, Wei Y, Liang K, Wang L, Zhang S (2021). Facile in-situ deposition of Pt nanoparticles on nano-pore stainless steel composite electrodes for high active hydrogen evolution reaction. Int. J. Hydrogen Energy.

[11] Atchudan R (2022). Facile synthesis of novel molybdenum disulfide decorated banana peel porous carbon electrode for hydrogen evolution reaction. Chemosphere.

[12] Padmapriya S, Harinipriya S (2020). Electrocatalytic performance of polypyrrole synthesized using different oxidants and its hydrogen storage ability. Solid State Technol..

[13] Ashassi-Sorkhabi H, Kazempour A (2020). Incorporation of organic/inorganic materials into polypyrrole matrix to reinforce its anticorrosive properties for the protection of steel alloys: A review. J. Mol. Liq..

[14] Rabl H (2020). Are polyaniline and polypyrrole electrocatalysts for oxygen (O_2_) reduction to hydrogen peroxide (H_2_O_2_)?. ACS Appl. Energy Mater..

[15] Malinauskas A (1999). Electrocatalysis at conducting polymers. Synth. Met..

[16] Ramohlola KE (2018). Polyaniline-metal organic framework nanocomposite as an efficient electrocatalyst for hydrogen evolution reaction. Compos. B Eng..

[17] Ashassi-Sorkhabi H (2022). Electrodes derived from conducting polymers and their composites for catalytic conversion of carbon dioxide. J. Electrochem. Soc..

[18] Vinodh R (2020). Novel 13X Zeolite/PANI electrocatalyst for hydrogen and oxygen evolution reaction. Int. J. Hydrogen Energy.

[19] Madaswamy SL (2021). Polyaniline-based nanocomposites for direct methanol fuel cells (DMFCs)-A recent review. J. Ind. Eng. Chem..

[20] Mirzaei-Saatlo, M., Asghari, E., Shekaari, H., Pollet, B. G. & Vinodh, R. Performance of ethanolamine-based ionic liquids as novel green electrolytes for the electrochemical energy storage applications. *Electrochim. Acta*, 143499 (2023).

[21] Attar A (2022). Fabrication, characterization, TD-DFT, optical and electrical properties of poly (aniline-co-para nitroaniline)/ZrO_2_ composite for solar cell applications. J. Ind. Eng. Chem..

[22] Al-Hossainy AF, Zoromba MS (2019). Doped-poly (para-nitroaniline-co-aniline): Synthesis, semiconductor characteristics, density, functional theory and photoelectric properties. J. Alloys Compd..

[23] Rashid M, Sabir S (2009). Oxidative copolymerization of aniline with o-and p-nitroaniline by ammonium per sulfate: Kinetic and pathway. J. Dispers. Sci. Technol..

[24] Hussein HF (2014). Preparation of poly (aniline-co-p-nitro aniline) by spin-coating and study of the effect of thickness on energy gap. Walailak J. Sci. Technol..

[25] Arjomandi J, Makhdomi H, Parvin MH (2016). Novel conducting poly (p-nitro aniline-co-N-methyl aniline): Electrosynthesis, mechanism and in situ spectroelectrochemical characterization. Synth. Met..

[26] Cai J (2013). Fabrication of three-dimensional nanoporous nickel films with tunable nanoporosity and their excellent electrocatalytic activities for hydrogen evolution reaction. Int. J. Hydrogen Energy.

[27] Darband GB, Aliofkhazraei M, Rouhaghdam AS (2018). Three-dimensional porous Ni-CNT composite nanocones as high performance electrocatalysts for hydrogen evolution reaction. J. Electroanal. Chem..

[28] Hüner B, Demir N, Kaya MF (2023). Ni-Pt coating on graphene based 3D printed electrodes for hydrogen evolution reactions in alkaline media. Fuel.

[29] Ashassi-Sorkhabi, H., Kazempour, A., Moradi-Alavian, S., Asghari, E. & Lamb, J. J. 3D nanostructured nickel film supported to a conducting polymer as an electrocatalyst with exceptional properties for hydrogen evolution reaction. *Int. J. Hydrogen Energy* **48**, 29865–29876 (2023).

[30] Jayaseelan C (2014). Effect of sub-acute exposure to nickel nanoparticles on oxidative stress and histopathological changes in *Mozambique*
*tilapia, Oreochromis mossambicus*. Ecotoxicol. Environ. Saf..

[31] Taghizadeh F (2016). The study of structural and magnetic properties of NiO nanoparticles. Opt. Photonics J..

[32] Darabdhara G (2015). Reduced graphene oxide nanosheets decorated with Au, Pd and Au–Pd bimetallic nanoparticles as highly efficient catalysts for electrochemical hydrogen generation. J. Mater. Chem. A.

[33] Wu L (2022). Efficient alkaline water/seawater hydrogen evolution by a nanorod-nanoparticle-structured Ni-MoN catalyst with fast water-dissociation kinetics. Adv. Mater..

[34] Cai W (2018). Ni5P4-NiP2 nanosheet matrix enhances electron-transfer kinetics for hydrogen recovery in microbial electrolysis cells. Appl. Energy.

[35] Majhi KC, Yadav M (2021). Sphere-shaped bimetallic sulphoselenide: An efficient electrocatalyst for hydrogen evolution reaction. Energy Fuels.

[36] Majhi KC, Yadav M (2021). Bimetallic chalcogenide nanocrystallites as efficient electrocatalyst for overall water splitting. J. Alloys Compd..

[37] Majhi KC, Yadav M (2021). Palladium oxide decorated transition metal nitride as efficient electrocatalyst for hydrogen evolution reaction. J. Alloys Compd..

[38] Majhi KC, Yadav M (2020). Transition metal chalcogenides based nanocomposites as efficient electrocatalyst for hydrogen evolution reaction over the entire pH range. Int. J. Hydrogen Energy.

[39] Tian F (2021). Interface engineering: PSS-PPy wrapping amorphous Ni-Co-P for enhancing neutral-pH hydrogen evolution reaction performance. Chem. Eng. J..

[40] Hu G (2019). Enhanced electrocatalytic activity of WO3@ NPRGO composite in a hydrogen evolution reaction. Appl. Surf. Sci..

[41] Dong Y, Feng J, Li G (2017). Transition metal ion-induced high electrocatalytic performance of conducting polymer for oxygen and hydrogen evolution reactions. Macromol. Chem. Phys..

[42] Lahiri A, Li G, Endres F (2020). Highly efficient electrocatalytic hydrogen evolution reaction on carbonized porous conducting polymers. J. Solid State Electrochem..

[43] Dalla Corte DA, Torres C, dos Santos Correa P, Rieder ES, de Fraga Malfatti C (2012). The hydrogen evolution reaction on nickel-polyaniline composite electrodes. Int. J. Hydrogen Energy.

[44] Ma Y-Y (2017). Highly efficient hydrogen evolution from seawater by a low-cost and stable CoMoP@ C electrocatalyst superior to Pt/C. Energy Environ. Sci..

[45] Bisquert J, Randriamahazaka H, Garcia-Belmonte G (2005). Inductive behaviour by charge-transfer and relaxation in solid-state electrochemistry. Electrochim. Acta.

[46] Deng Y, Lai W, Xu B (2020). A mini review on doped nickel-based electrocatalysts for hydrogen evolution reaction. Energies.

[47] Seifzadeh D, Golmoghani-Ebrahimi E (2012). Formation of novel and crack free nanocomposites based on sol gel process for corrosion protection of copper. Surf. Coat. Technol..

[48] Córdoba-Torres P (2012). On the intrinsic coupling between constant-phase element parameters α and Q in electrochemical impedance spectroscopy. Electrochim. Acta.

[49] Amokrane N, Gabrielli C, Nogueira RP (2007). Indirect identification of Hads relaxation on different metals by electrochemical impedance spectroscopy. Electrochim. Acta.

[50] Sanabria H, Miller JH (2006). Relaxation processes due to the electrode-electrolyte interface in ionic solutions. Phys. Rev. E.

[51] Solomon G (2019). Ag2S/MoS2 nanocomposites anchored on reduced graphene oxide: Fast interfacial charge transfer for hydrogen evolution reaction. ACS Appl. Mater. Interfaces.

[52] Cai H (2022). N-doped CNT as electron transport promoter by bridging CoP and carbon cloth toward enhanced alkaline hydrogen evolution. Chem. Eng. J..

[53] Tsai F-T (2020). The HER/OER mechanistic study of an FeCoNi-based electrocatalyst for alkaline water splitting. J. Mater. Chem. A.

[54] Ashassi-Sorkhabi H, Kazempour A, Moradi-Alavian S, Asghari E, Lamb JJ (2023). 3D nanostructured nickel film supported to a conducting polymer as an electrocatalyst with exceptional properties for hydrogen evolution reaction. Int. J. Hydrogen Energy.

[55] Cossar E, Houache MSE, Zhang Z, Baranova EA (2020). Comparison of electrochemical active surface area methods for various nickel nanostructures. J. Electroanal. Chem..

[56] Bakovic SIP (2021). Electrochemically active surface area controls HER activity for FexNi100−x films in alkaline electrolyte. J. Catal..

[57] McCrory CCL (2015). Benchmarking hydrogen evolving reaction and oxygen evolving reaction electrocatalysts for solar water splitting devices. J. Am. Chem. Soc..

[58] McCrory CCL, Jung S, Peters JC, Jaramillo TF (2013). Benchmarking heterogeneous electrocatalysts for the oxygen evolution reaction. J. Am. Chem. Soc..

[59] Ashassi-Sorkhabi H, Moradi-Alavian S, Esrafili MDMD, Kazempour A (2019). Hybrid sol-gel coatings based on silanes-amino acids for corrosion protection of AZ91 magnesium alloy: Electrochemical and DFT insights. Prog. Org. Coat..

[60] Niu X, Tang Q, He B, Yang P (2016). Robust and stable ruthenium alloy electrocatalysts for hydrogen evolution by seawater splitting. Electrochim. Acta.

[61] Sun Y (2021). Biomimetic assembly to superplastic metal–organic framework aerogels for hydrogen evolution from seawater electrolysis. Exploration.

